# Supplementary material for: DJ-1 controls bone homeostasis through the regulation of osteoclast differentiation
Source: Nat Commun. 2017 Nov 15;8:1519. doi: 10.1038/s41467-017-01527-y (PMC5688089; doi:10.1038/s41467-017-01527-y)
Supplement: Supplementary file 1 — Supplementary Information [file 41467_2017_1527_MOESM1_ESM.pdf]

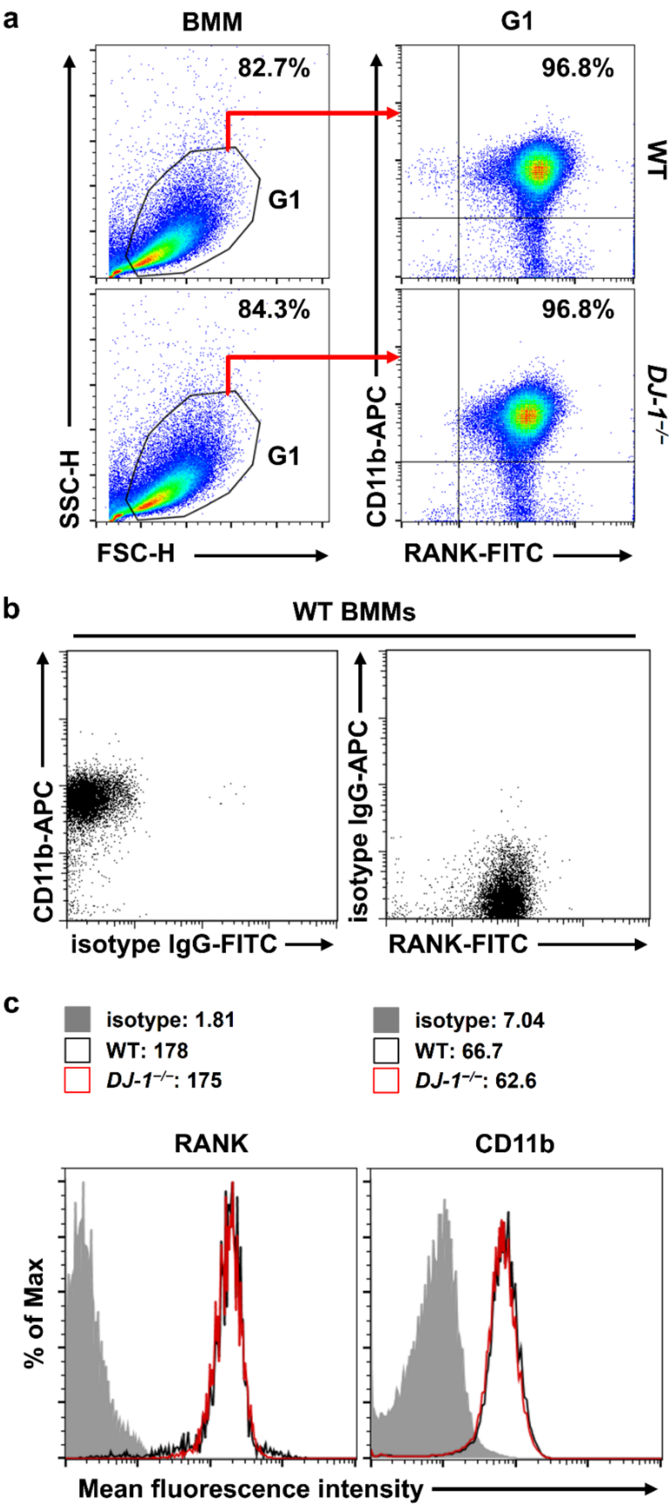
a
BMM
G1
82.7%
84.3%
G1
G1
96.8%
96.8%
WT
DJ-1−/−
SSC-H
FSC-H
CD11b-APC
RANK-FITC
b
WT BMMs
CD11b-APC
isotype IgG-FITC
isotype IgG-APC
RANK-FITC
c
isotype: 1.81
WT: 178
DJ-1−/−: 175
isotype: 7.04
WT: 66.7
DJ-1−/−: 62.6
RANK
CD11b
% of Max
Mean fluorescence intensity

Supplementary Figure 1. Comparison of RANK expression in WT and *DJ-1*$^{-/-}$ BMMs. (**a**) Flow cytometric analysis for RANK in BMMs from WT and *DJ-1*$^{-/-}$ mice. After gating for monocytes (G1), and RANK expressing BMMs were identified by CD11b and RANK Abs. (**b**) Flow cytometric analysis of expression of RANK and CD11b in BMMs from WT and *DJ-1*$^{-/-}$ mice against each isotype control. (**c**) Mean fluorescence intensity (MFI) for RANK and CD11b expression on BMMs from WT and *DJ-1*$^{-/-}$ mice. All representative images from three independent experiments.

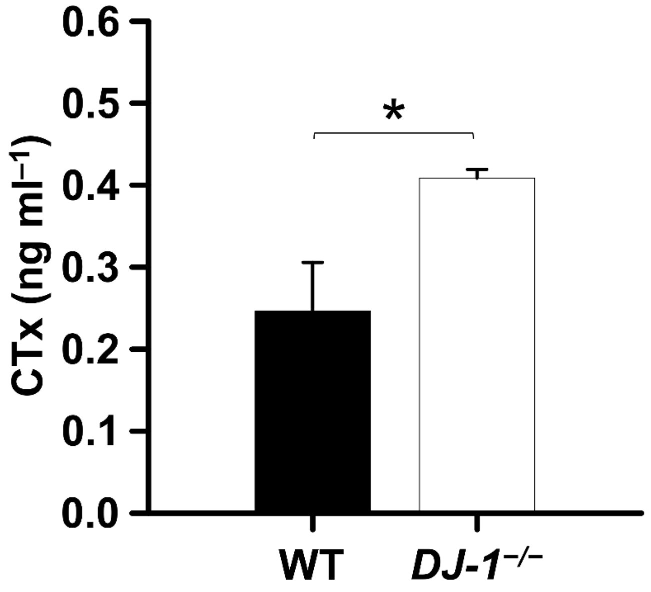

Supplementary Figure 2. Increased levels of collagen type I fragment (CTx) in RANKL stimulated *DJ-1*$^{-/-}$ BMMs in vitro. (**a**) Measurement of CTx in media from cultures of BMMs derived from WT and *DJ-1*$^{-/-}$ mice 4 days after RANKL stimulation. Data shown as mean ± s.e.m. from three independent experiments in triplicate. **p < 0.05 by Student's t-test.

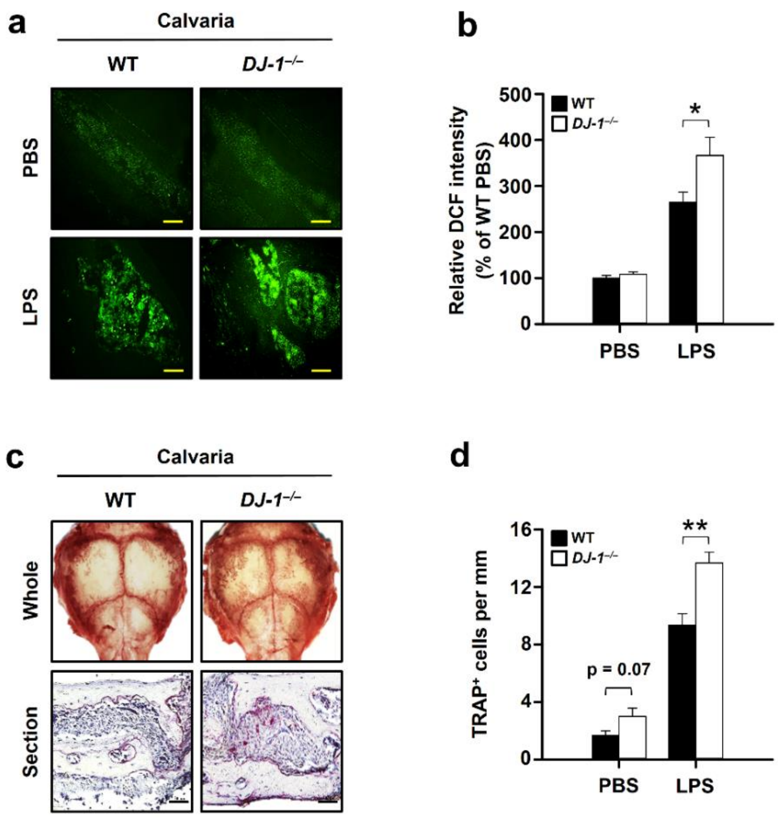

Supplementary Figure 3. DJ-1 deficiency causes the increase of ROS amount and the number of OCs in LPS-induced bone loss model. The mice were injected with 25 mg/kg of LPS in PBS subcutaneously over the calvaria for 7 days. (**a**) Representative images for DCF fluorescence and (**b**) relative DCF intensities in LPS-stimulated calvaria in WT and *DJ-1*$^{-/-}$ mice. Scale bar, 50 μm; $n = 5$ mice. *$p < 0.05$ versus WT by Student's *t*-test. (**c**) Representative images for TRAP-stained whole (upper) and sectioned (lower) calvaria tissues in LPS-treated WT and *DJ-1*$^{-/-}$ mice. Scale bar, 50 μm. (**d**) Number of TRAP$^+$ cells in cavaria tissues in LPS-injected WT and *DJ-1*$^{-/-}$ mice; $n = 5$ per group. **$p < 0.01$ versus WT by Student's *t*-test. Representative images (**a,c**) or the mean ± s.e.m. (**b,d**) are shown.

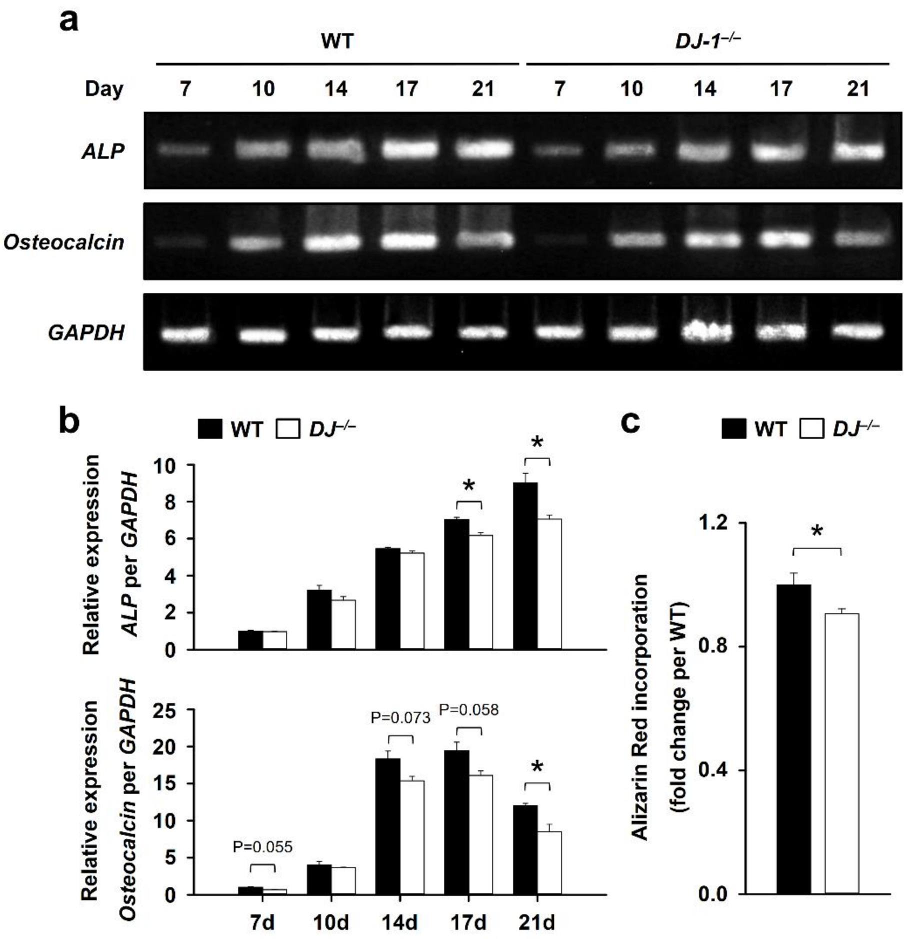

Supplementary Figure 4. Differentiation of osteoblastic stromal cells in WT and *DJ-1*$^{-/-}$ mice. (**a**) Representative images for mRNA of *alkaline phosphate (ALP)* and *osteocalcin* in WT or *DJ-1*$^{-/-}$ osteoblastic stromal cells. (**b**) Band density for panel a; *n* = 4 cells. (**c**) Measurement of mineralization using Alizarin red S; *n* = 4 cells. Representative images (**a**) or the mean ± s.e.m. (**b,c**) from three independent experiments in triplicate are shown. *p < 0.05 versus WT by Student's *t*-test.

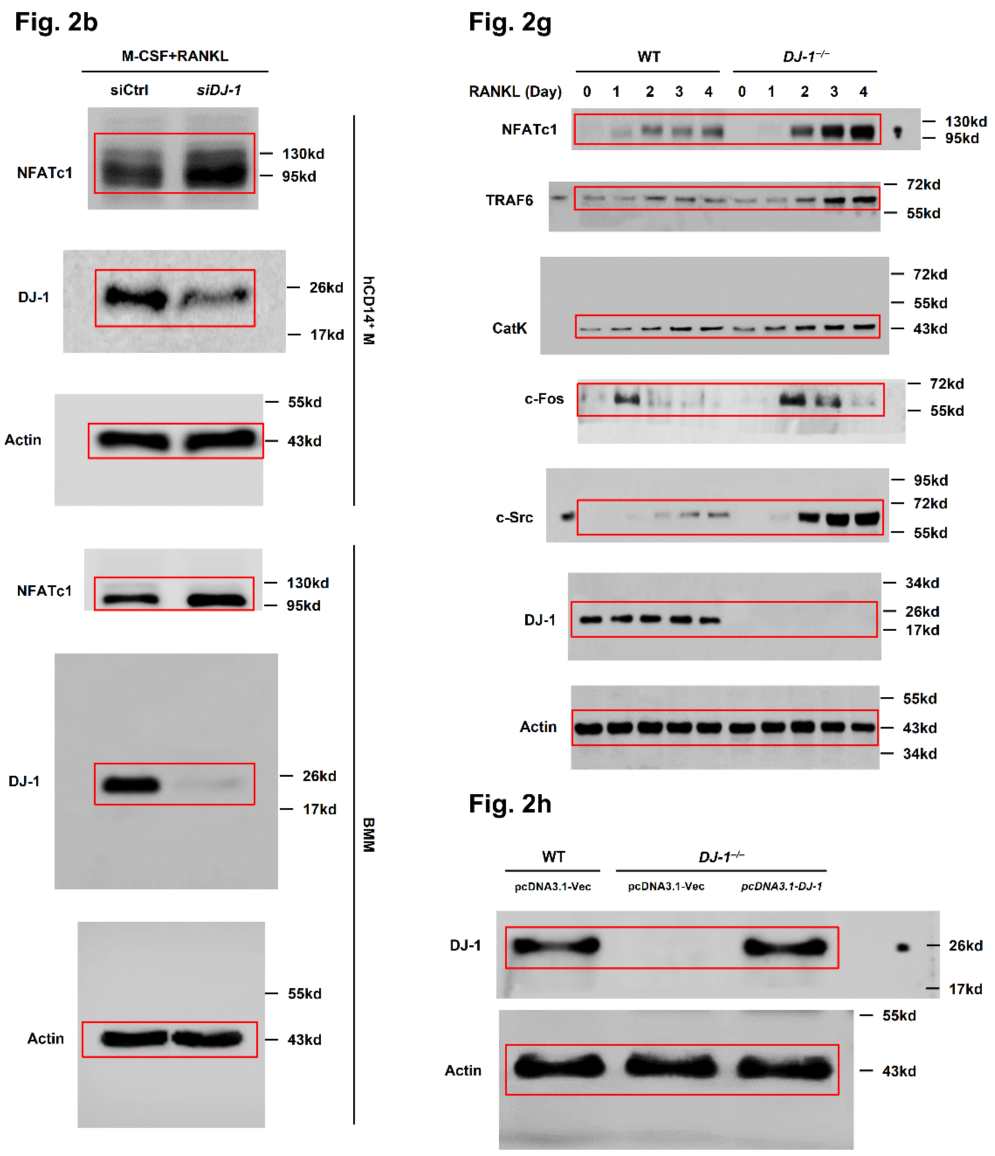

Supplementary Figure 5. Uncropped images of immunoblot data for figure 2b-2h.

Fig. 3a

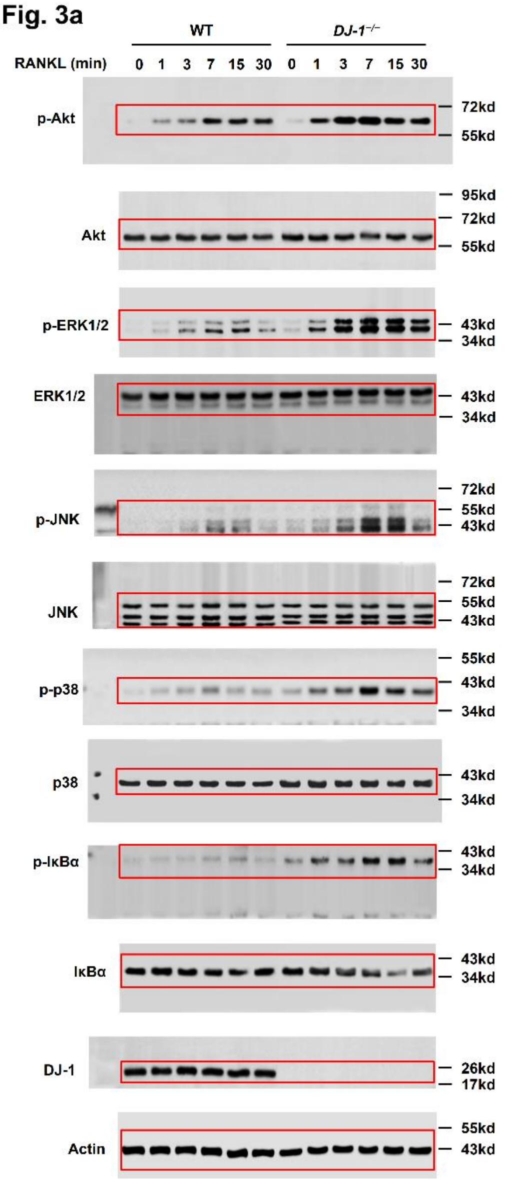

Fig. 3b

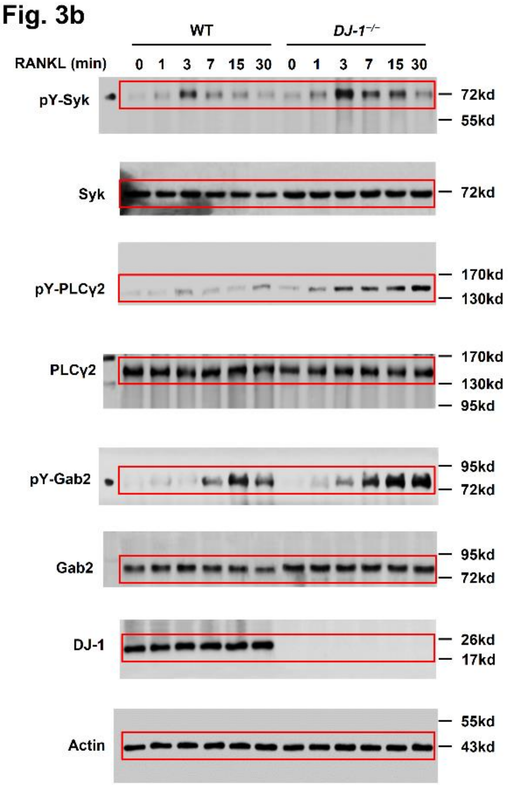

Supplementary Figure 6. Uncropped images of immunoblot data for figure 3a-3b.

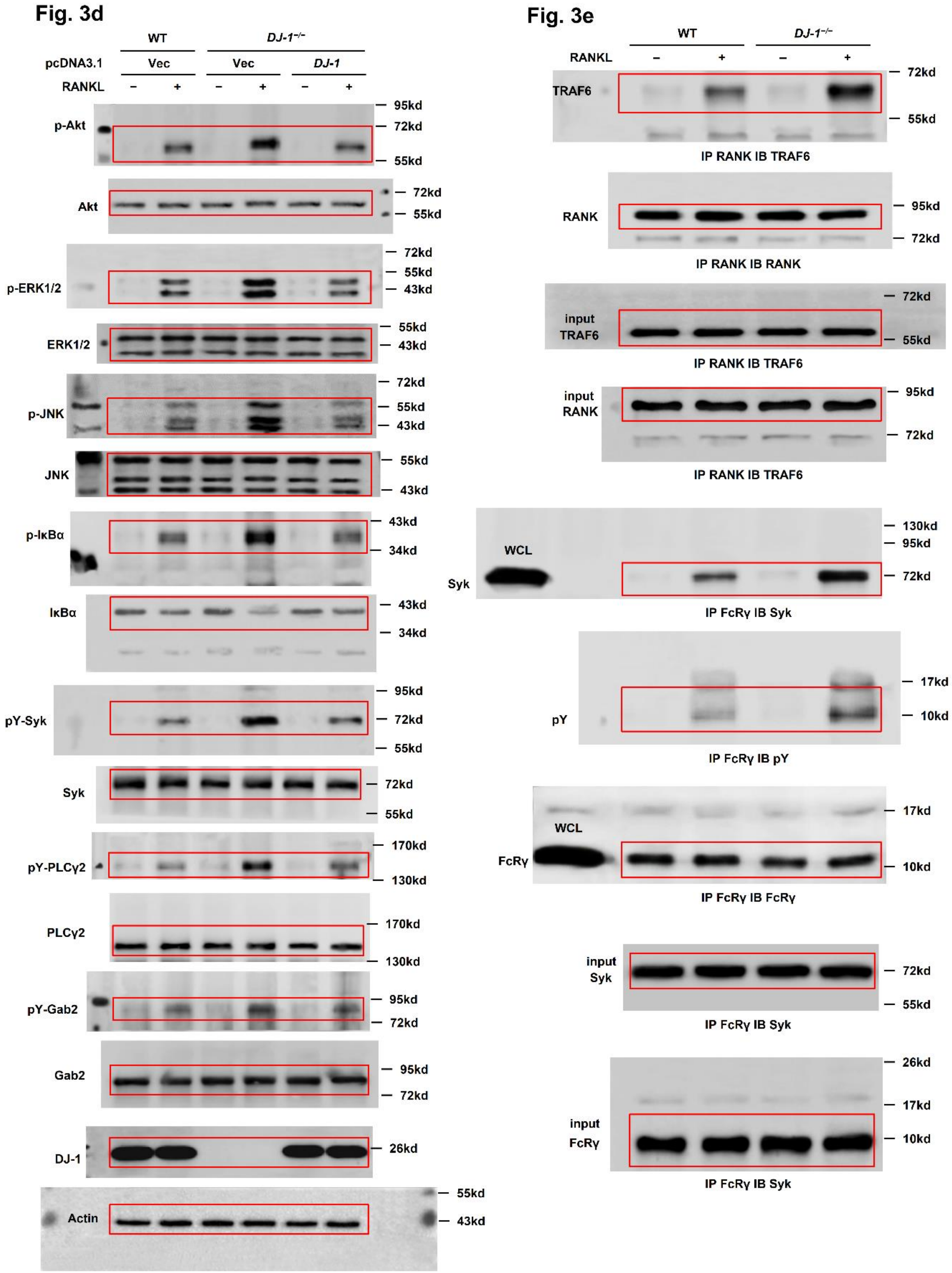

Supplementary Figure 7. Uncropped images of immunoblot data for figure 3d-3e.

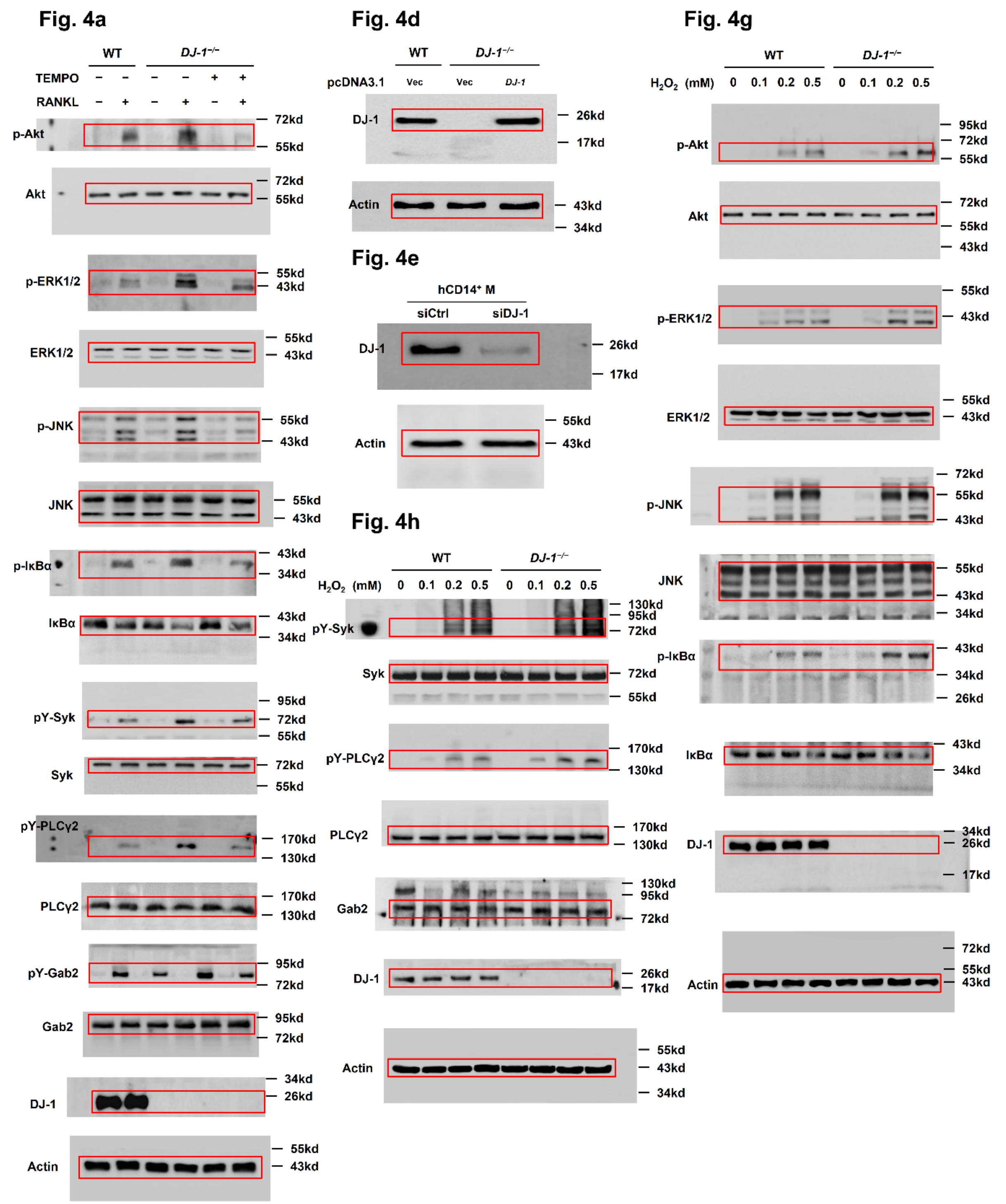

Supplementary Figure 8. Uncropped images of immunoblot data for figure 4a-4g.

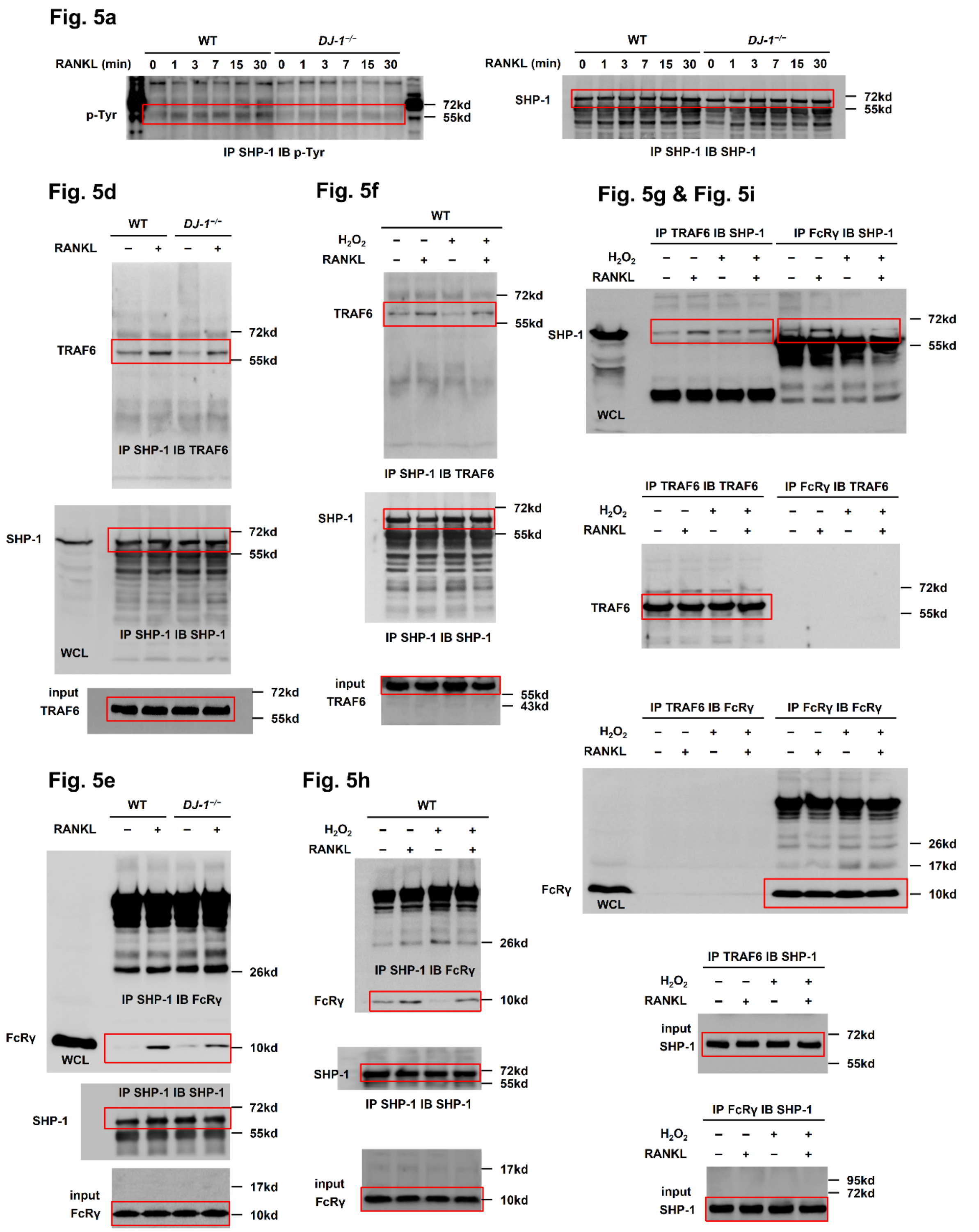

Supplementary Figure 9. Uncropped images of immunoblot data for figure 5a-5i.

Fig. 7h

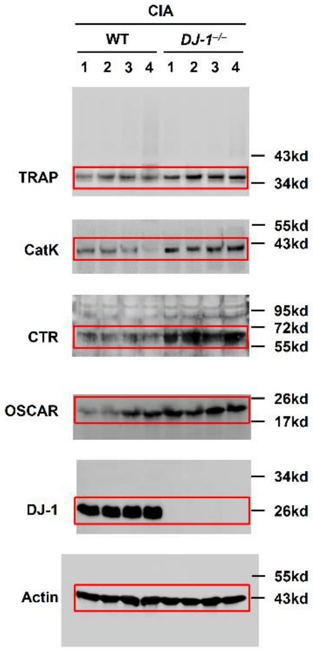

Supplementary figure 4a

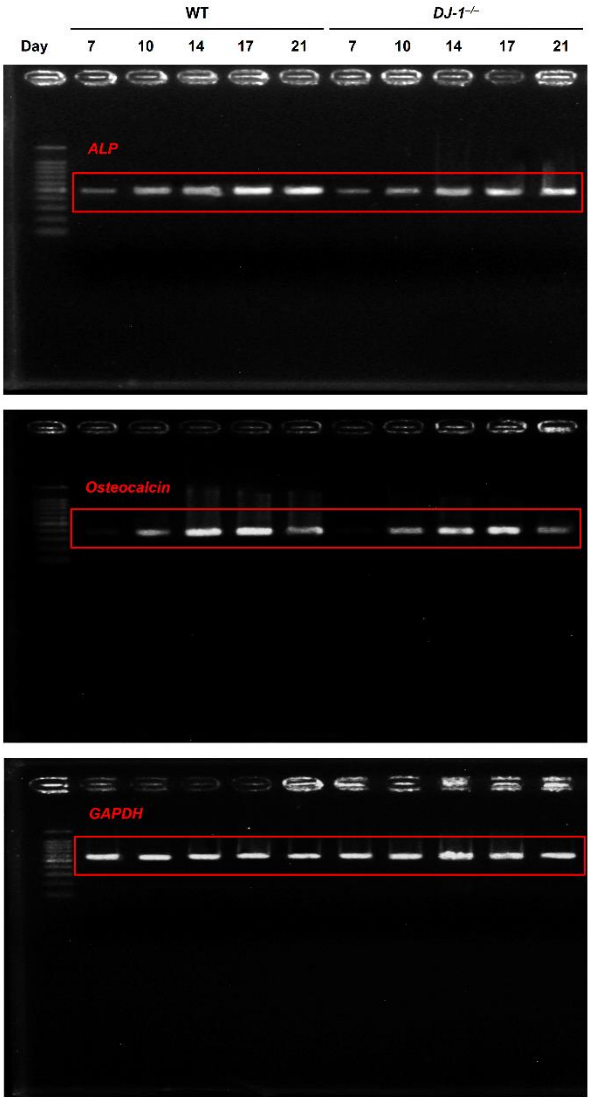

Supplementary Figure 10. Uncropped images of immunoblot data for figure 7h and supplementary figure 4a.
